# Far-field optical imaging of surface plasmons with a subdiffraction limited separation

**DOI:** 10.1515/nanoph-2020-0500

**Published:** 2020-12-18

**Authors:** Yifeng Xiang, Junxue Chen, Xi Tang, Ruxue Wang, Qiwen Zhan, Joseph R. Lakowicz, Douguo Zhang

**Affiliations:** Key Laboratory of OptoElectronic Science and Technology for Medicine of Ministry of Education, Fujian Provincial Key Laboratory of Photonics Technology, College of Photonic and Electronic Engineering, Fujian Normal University, Fuzhou 350117, China.; College of Science, Guilin University of Technology, Guilin, 541004, China; Department of Optics and Optical Engineering, Institute of Photonics, University of Science and Technology of China, Hefei, Anhui, 230026, China; State Key Laboratory of Functional Materials for Informatics, Shanghai Institute of Microsystem and Information Technology, Chinese Academy of Sciences, Shanghai, 200050, China; Department of Electro-Optics and Photonics, University of Dayton, 300 College Park, Dayton, OH, 45469-2951, USA; School of Optical-Electrical and Computer Engineering, University of Shanghai for Science and Technology, Shanghai, 200093, China.; Department of Biochemistry and Molecular Biology, Center for Fluorescence Spectroscopy, University of Maryland School of Medicine, 725 West Lombard St., Baltimore, MD, 21201, USA; Department of Optics and Optical Engineering, Institute of Photonics, University of Science and Technology of China, Hefei, Anhui, 230026, China

**Keywords:** diffraction limit, leakage radiation microscopy, photonic band gap, silver nanowire, surface plasmon

## Abstract

When an ultrathin silver nanowire with a diameter less than 100 nm is placed on a photonic band gap structure, surface plasmons can be excited and propagate along two side-walls of the silver nanowire. Although the diameter of the silver nanowire is far below the diffraction limit, two bright lines can be clearly observed at the image plane by a standard wide-field optical microscope. Simulations suggest that the two bright lines in the far-field are caused by the unique phase distribution of plasmons on the two side-walls of the silver nanowire. Combining with the sensing ability of surface plasmons to its environment, the configuration reported in this work is capable of functioning as a sensing platform to monitor environmental changes in the near-field region of this ultrathin nanowire.

## 1 Introduction

Surface plasmons (SPs) can confine light into subwavelength spatial dimensions, which makes them promising for use in nanophotonic devices [1, 2]. Silver nanowires (Ag NWs) synthesized using wet-chemistry approaches are particular useful for constructing plasmonic waveguides due to their subwavelength diameters, high refractive indices, atomic surface smoothness and large aspect ratios[3–5]. The light can be coupled into Ag NWs as propagating SPs with the electric field tightly confined at the metal surface. Until now, Ag NWs have been widely used for development of nanophotonic integrated circuits [6, 7], and several functionalities have been realized, such as plasmonic routers [8], and logic gates [9, 10].

Because Ag NWs are very thin and soft, they are always placed on solid substrates for practical applications. The SPs propagating along Ag NWs can be detected when the photon flux into the substrate is collected and imaged in the far-field by an optical system. When Ag NWs are placed on a substrate, there are two kinds of supported modes: a bound mode whose effective refractive index is larger than that of the substrate and a leaky mode whose effective refractive index is smaller than that of the substrate. For the plasmonic bound mode, the electric field is localized at the nanowire/glass interface that cannot leak photons into the substrate. Only the scattered light in the end of Ag NWs can be collected and imaged by the optical system [7, 8]. Quantum dot fluorescence is often used to image the electric field distribution of the propagating SPs on Ag NWs [9–11]. However, the electric field localized at the two side-walls of Ag NWs is a leaky mode, which can couple to photons in the substrate and be directly imaged in the far-field. The far-field imaging of SPs propagating along the two side-walls of an Ag NW shows two bright lines [12, 13]. Because these reports on plasmonic leaky modes are associated with thick Ag NWs and their diameters are larger than the diffraction limit, such as diameters larger than 300 nm or even larger, this phenomenon does not attract much attention.

Our previous work demonstrated ultrathin Ag NWs (90nm) placed on a photonic band gap (PBG) structure could have a plasmonic leaky mode with the plasmons propagating along the two side-walls of the Ag NWs [14]. Although their distance in the near-field is far below the diffraction limit, two bright lines were observed at the image plane in the far-field by a standard wide-field optical microscope. However, the mechanism of the far-field optical imaging with a subdiffraction limited separation is not clear. In this article, the far-field optical imaging of SPs propagating along Ag NWs with diameters of 60 and 40 nm is realized. We find the distance between the two bright lines in the image plane is not related to the diameter of the Ag NWs, but to the imaging system. Simulations suggest that the two lines in the far-field are due to the unique phase distribution of the SPs on the two side-walls of the Ag NW. Because of the sensitivity of plasmonic field to the local environment, this configuration can work as a sensing platform to monitor the environmental changes in the near-field region of ultrathin Ag NWs.

## 2 Results and discussion

### 2.1 Far-field imaging of SPs propagating along an Ag NW on a PBG structure

**Figure 1:**
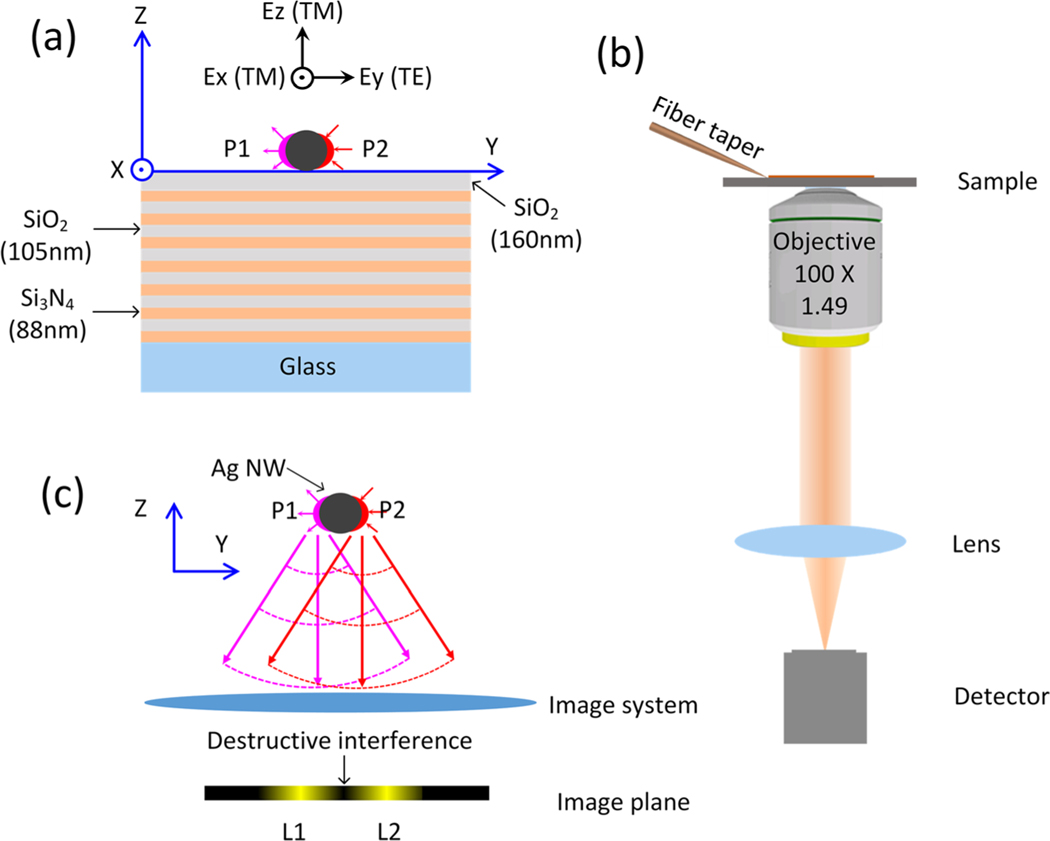
Schematic illustration of the experiment. (a) An Ag NW is placed on a dielectric multilayer consisting of alternating layer of SiO_2_ (105 nm thick) and Si_3_N_4_ (88 nm thick). There are 14 layers in total, with a top SiO_2_ layer with a thickness of 170 nm. Ex and Ez components are TM polarization to the substrate, and Ey component is TE polarization to the substrate. P1 and P2 represent the SPs propagating along the two side-walls of the Ag NW. (b) Optical setup of the leakage radiation microscope system. A fiber taper is used to couple the laser beam into the Ag NW. A detector is used for capturing the image plane. (c) Illustration of imaging for P1 and P2. There is a *π*-phase difference between P1 and P2, and then the destructive interference happens at the image plane.

Figure 1 shows the schematic illustration of the platform containing an Ag NW on a dielectric multilayer substrate, which is a PBG structure. The planar PBG structure has 14 layers and the thickness of each layer is shown in Figure 1(a). Except for the top SiO_2_ layer with thickness of 160 nm, the thickness of SiO_2_ and Si_3_N_4_ layers are 105 and 88 nm, respectively. Here, SiO_2_ is the low refractive index dielectric (*n*_SiO2_ =1.46) and Si_3_N_4_ is the high refractive index dielectric (*n*_Si3N4_ = 2.14). The refractive index of the glass substrate is 1.515. By using these structural parameters, a PBG will be generated at the incident wavelength of 590 nm [15,16], which plays a key role in our method. If an ultrathin Ag NW is placed on a glass substrate, the plasmonic leaky mode disappears and the SPs cannot propagate along the two side-walls of this Ag NW for light in the visible regime due to the electric field mostly dissipating into the glass substrate [17]. In this case, only the plasmonic bound mode exists (Figure S1 in Supplementary Material). However, if an ultrathin Ag NW is placed on the appropriately designed PBG structure, the forbidden band will prevent the dissipation of the SPs into the substrate and reduce the radiative energy loss, which can sustain the SPs propagating along the two side-walls of the Ag NW [14]. A Similar phenomenon also occurs with dielectric nanofibers [18].

The optical configuration for this experiment is presented in Figure 1(b). It is a home-built wide-field microscope with an oil-immersion objective, which is also referred to as a leaky radiation microscopy (LRM) because the imaging is based on the leaky radiation signals from SPs or Bloch surface waves (BSWs) [12, 19]. A single straight Ag NW made with wet-chemistry methods was placed on this PBG structure. The SPs propagating along the Ag NW could be excited with the aid of a fiber taper that was placed very close to one end or a side-wall of the Ag NW when the incident wavelength was 590 nm. During its propagation, the SPs on every point of the Ag NW leak photons into the substrate, which can be imaged by a far-field detector equipped on the LRM. The intensity of the leaky radiation signal, at a given position of the Ag NW, is proportional to that of the SPs at the same position [20, 21]. The observation of the leaky radiation gives a direct measurement of the SPs traveling along the Ag NW.

**Figure 2:**
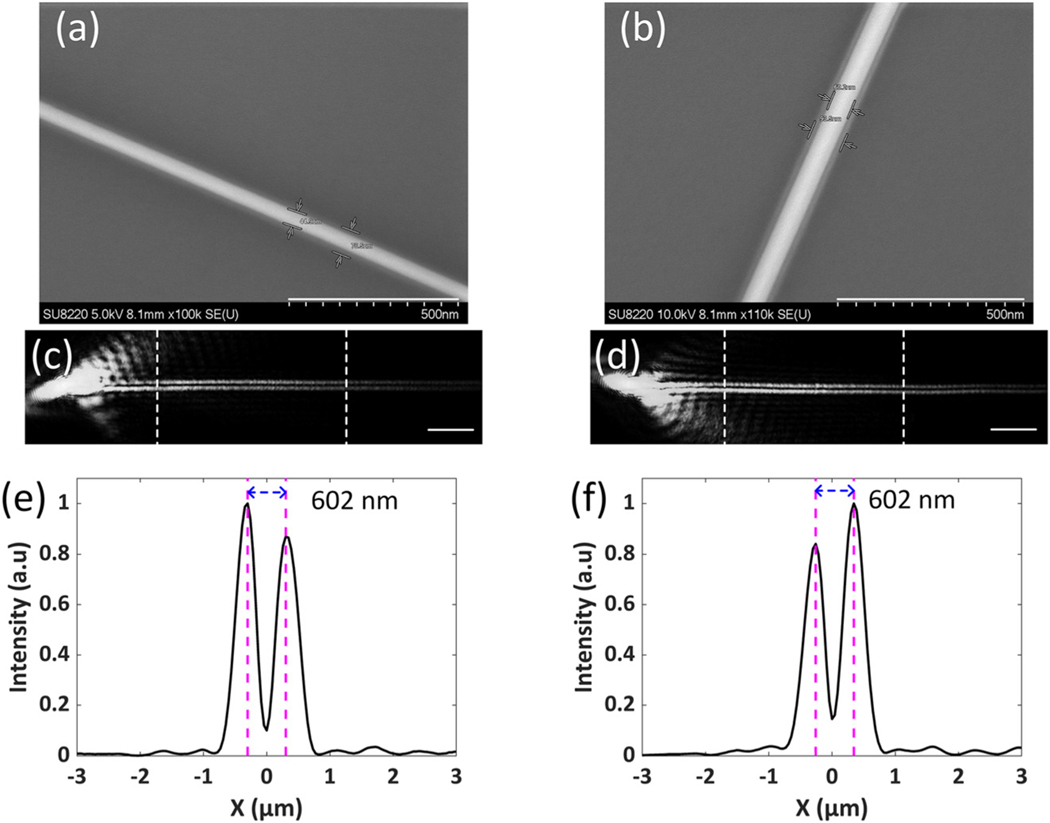
The images of SPs propagating along an Ag NW in the far-field. The diameter of the Ag NW is about 40 nm in (a, c, e), and is about 60 nm in (b, d, f). (a, b) SEM images of the representative Ag NWs. The length of scale bars is 500 nm. Ag NWs are coated with a polymer cladding layer (thickness of about 15 nm). (c, d) The images of the SPs propagating along an Ag NW. The length of scale bars is 5 μm. (e, f) The corresponding average intensity profiles perpendicular to the Ag NW in the area between the white dashed lines in (c, d). The distance between two peaks is 602 nm in both cases.

In our experiment, the diameters of Ag NWs deposited on the PBG structure are about 40 and 60 nm, as seen in Figure 2(a) and (b). The scanning electronic microscope (SEM) images show that the Ag NWs are coated with a polymer (polyvinyl pyrrolidone, PVP) cladding layer with a thickness of about 15 nm, which can prevent the Ag NWs from oxidization and make them stable for long-term use. In Figure 2(c) and (d), two bright lines appear on the images of the SPs travelling along the Ag NW, which means there are two plasmonic fields propagating parallel to each other along this straight Ag NW. Figure 2(e) and (f) shows the intensity profiles perpendicular to the Ag NWs with diameters of 40 and 60 nm, respectively. The distance between the two bright lines at the image plane is 602 nm in both cases, which demonstrates that the distance between the two bright lines in the far-field is not proportional to the diameter of the Ag NW.

### 2.2 Numerical simulation of the plasmonic mode

**Figure 3:**
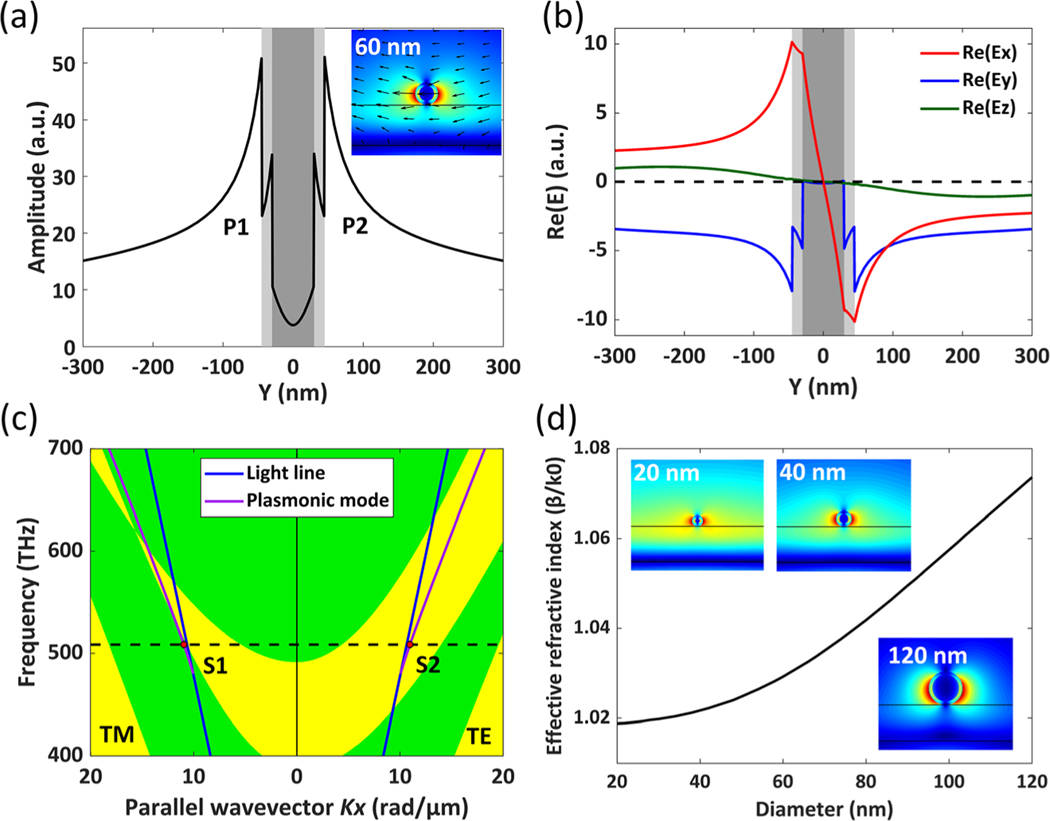
Numerical simulation of the plasmonic mode. (a) The profile of the electric field amplitude (|*E*|) across the center of the Ag NW. This inset graph shows the electric field amplitude distribution around the AgNWwith a diameter of 60 nm. The black arrows in the inset graphs denote the direction of the electric field vector. (b) The profiles of the real part for three electric field components (Ex, Ey and Ez) across the center of the Ag NW (inset graph on (a)). (c) The projected band structure of the dielectric multilayer for TE and TM polarizations. The yellow zone denotes the forbidden band. The green zone denotes the conduction band. The blue line denotes the light line in the air. The purple line denotes the dispersion relation for the plasmonic mode. The red spots (S1 and S2) on purple line correspond to the excitation wavelength at 590 nm. (d) The effective refractive index for the plasmonic mode versus the diameter of an Ag NW. The electric field amplitude distributions for the plasmonic mode of an Ag NW with diameters of 20, 40 and 120 nm are shown in the inset graphs.

To clearly demonstrate the mechanism of the optical imaging of the plasmons with a subdiffraction limited separation, numerical simulations of the plasmonic mode for an Ag NW placed on the PBG structure were performed. The electric field amplitude (|*E*|) distribution of the plasmonic mode (insetgraph on Figure 3(a)) shows that the plasmonic field can be divided into two parts (P1 and P2), which are located on the two sides of the Ag NW. The profile of the electric filed amplitude across the center of the Ag NW (inset graph on Figure 3(a)) is plotted in Figure 3(a), which shows the distance between the peaks of the two plasmonic fields is 90 nm, which is equal to the total diameter of the PVP-coated Ag NW and far below the diffraction limit. During the experiment, the incident wavelength is fixed at 590 nm and the numerical aperture (NA) of the objective is 1.49. Hence, the diffraction limited spatial separation that can be resolved by the imaging system should be no less than 242 nm (0.61 × 590/1.49 nm). However, our experimental results, as shown in Figure 2, demonstrate that two bright lines can be observed in the far-field although the two plasmonic fields are separated less than 100 nm.

The profiles of the real part for three electric field components (Ex, Ey and Ez) across the center of the Ag NW were plotted in Figure 3(b). From those profiles, we find that the value of Ex (and Ez) component on one side-wall of the Ag NW is opposite to that on the other side-wall and the values of Ey component on the two side-walls are the same. It means that Ex (and Ez) component on the two side-walls of the Ag NW is out-of-phase whereas Ey component is in-phase. Because it’s a leaky mode, the plasmonic fields will radiate into the substrate, which can be collected by an oil-immersed objective and imaged in the far-field using an optical system. However, due to the forbidden band, not all leakage radiation can transmit through the PBG structure and reach the image plane in the far-field. Figure 3(c) shows the projected band structure of the dielectric multilayer for transverse-electric (TE) and transverse-magnetic (TM) polarizations and the dispersion relation for the plasmonic mode. The red spots on the plasmonic mode (S1 and S2) correspond to the excitation wavelength at 590 nm. It is noted that S1 is in the conduction band for TM polarization and S2 is in the forbidden band for TE polarization, which means the leakage radiation of TM-polarized plasmons can transmit through the PBG structure whereas that of TE-polarized plasmons is inhibited. The schematic diagram of TE/TM polarization to the substrate is shown in Figure 1(a). Ex and Ez components are TM polarization to the substrate and Ey component is TE polarization to the substrate. Interestingly, due to the inhibition for the leakage radiation of Ey component (TE polarization) by the forbidden band, only the Ex and Ez components (TM polarization), which are out-of-phase on the two side-walls of the Ag NW, can transmit through the dielectric multilayer and be imaged in the far-field. The opposite phase of Ex and Ez components causes destructive interference at the image plane, as shown in Figure 1(c), which is the key for the optical imaging of the plasmons with a subdiffraction limited separation. When both TE and TM polarization are in the forbidden band, the leakage radiation cannot pass through the PBG structure and the SPs propagating along an Ag NW cannot be imaged in the far-field (Figure S2 in Supplementary Material).

The diameter-dependent dispersion relation of the plasmonic mode for an Ag NW on the PBG structure is shown in Figure 3(d). The plasmonic mode always exists when the diameter of the Ag NW is in the range of 20–120 nm. The effective refractive index of the plasmonic mode is lower than that of the glass substrate, which confirms this is a leaky mode. For Ag NWs with different diameters (20, 40 and 120 nm), the electric field amplitude distributions, as shown in the inset graphs on Figure 3(d), are similar to that for the Ag NW with a diameter of 60 nm, so two bright lines can also be observed at the image plane, which is verified in the experiment and shown in Figure 2.

### 2.3 Simulated far-field imaging of the SPs propagating along an Ag NW

**Figure 4:**
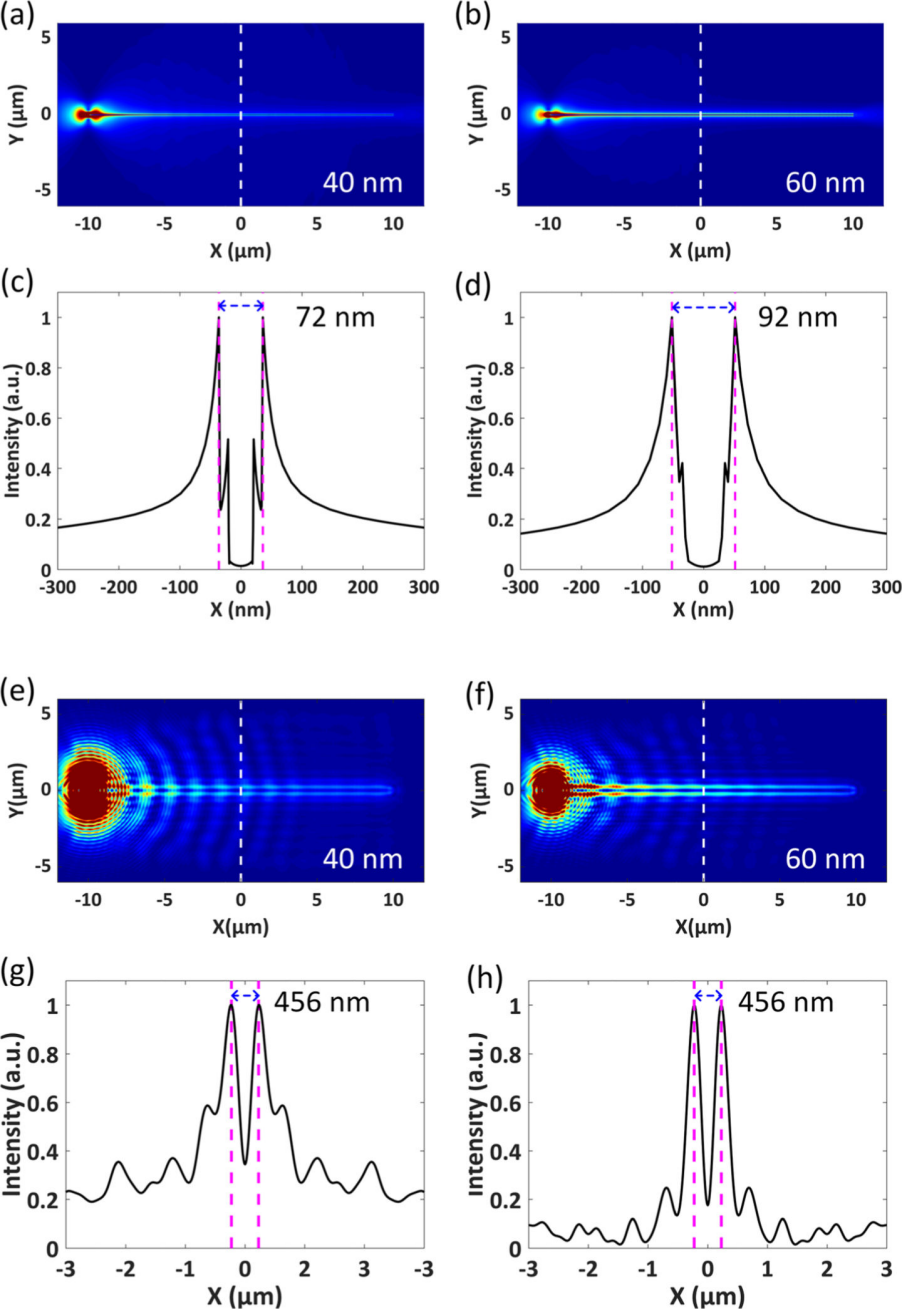
Simulated imaging of SPs propagating along an Ag NW. The diameter of an Ag NW is 40 nm in (a, c, e, g), and is 60 nm in (b, d, f, h). (a, b) The electric field intensity (|*E*|^2^) distributions at the *x-y* plane in the near-field. (c, d) The corresponding intensity profiles along the white dashed line in (a, b). The distance between two peaks is 72 nm in (c), and is 92 nm in (d). (e, f) The corresponding electric field intensity distributions at the image plane in (a, b). (g, h) The corresponding intensity profiles along the dashed white line in (e, f). The distance between two peaks is 456 nm in both cases.

To further verify the experimental results, the far-field imaging of SPs propagating along an Ag NW on the PBG structure was simulated. Figure 4(a) and (b) depicts the near-field distributions of the electric field intensity (|*E*|^2^) at the x-y plane for the Ag NW with diameters of 40 and 60 nm, respectively. When the incident light hits one end of the Ag NW, the SPs can be excited by the scattering light and propagate along the two side-walls of the Ag NW. The distance between the two plasmonic fields is 72 nm for the Ag NW with a diameter of 40 nm, as shown in Figure 4(c), and is 92 nm for the Ag NW with a diameter of 60 nm, as shown in Figure 4(d). From the electric field intensity distributions in the near-field, we can see that the SPs propagating along the two side-walls of the Ag NW are separated less than 100 nm, which is far below the diffraction limit.

The electric field intensity distributions at the image plane are given in Figure 4(e) and (f) when the sample plane is imaged in the far-field. Far-field photon flux into the substrate side was monitored and the corresponding electric field at the image plane was calculated by Fourier transformation of the far-field projection by taking into account the NA of the objective lens used in the experiment (*NA* = 1.49) (the detail is presented in Supplementary Material). The simulated images clearly show there are two bright lines at the image plane that are good agreement with the experimental observations in Figure 2. For the Ag NWs with diameters of 40 and 60 nm, the distance between the two lines in the image plane is 456 nm, which means the distance between the two lines is not related to the diameter of the Ag NWs. The simulated images in the far-field for different NA objectives are given in Figure S3 in Supplementary Material, which show that the distance between the two lines depends on the resolution ability of the imaging system. As the resolution of the imaging system increases, the distance between the two lines at the image plane decreases.

### 2.4 Sensing of the propagating SPs along an ultrathin Ag NW to the environment

**Figure 5:**
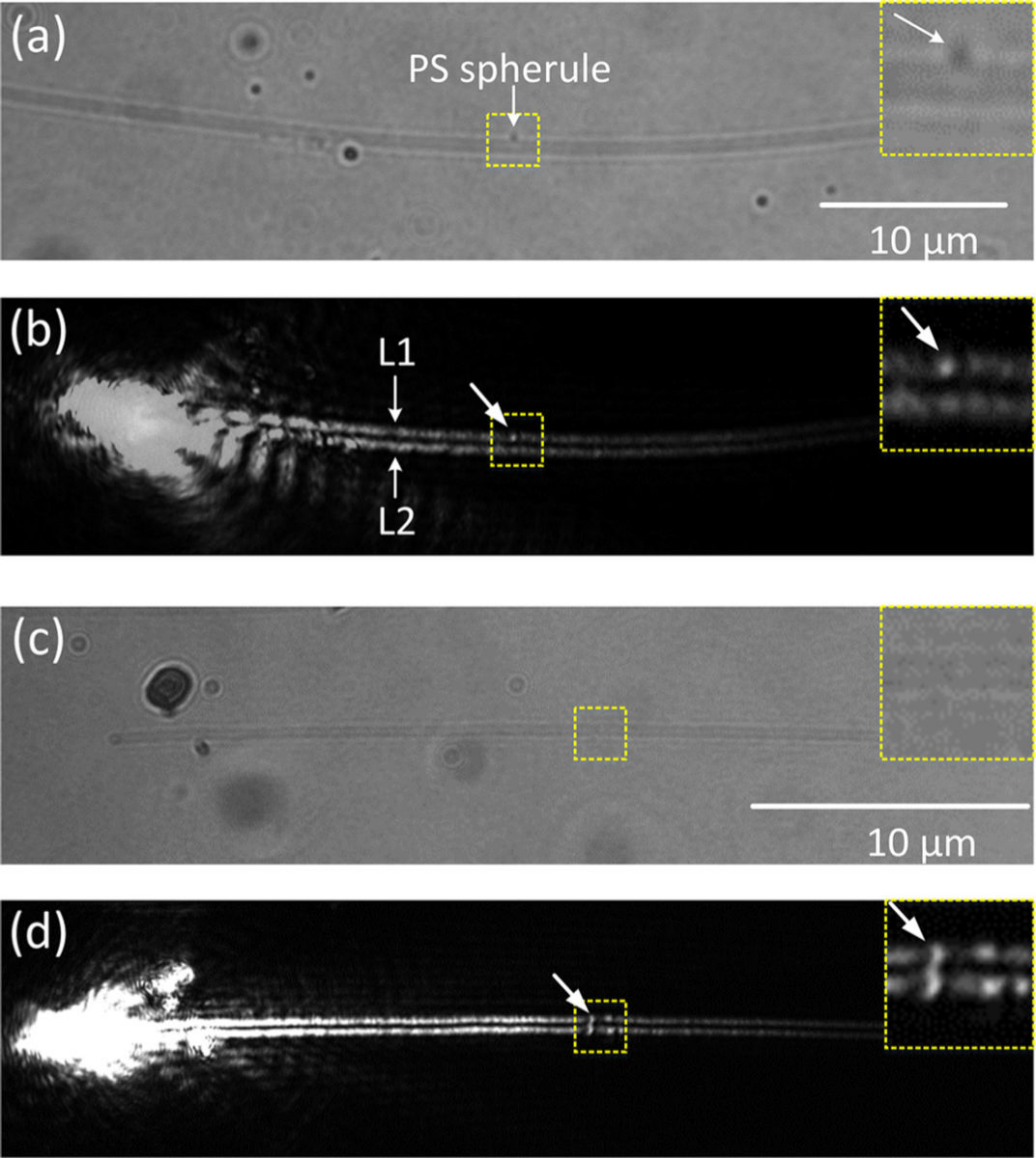
Sensing of the propagating SPs to environmental changes. (a, c) The bright-field images of the Ag NW. (b, d) The corresponding images of SPs propagating along the Ag NW for (a, c). On (a, b), the environmental change is induced by a PS nanoparticle attached on the top side-wall of the Ag NW. On (c, d), there is something on this Ag NW, which cannot be observed on the bright-field image (c), but can be observed on the plasmonic image (d), as shown in the area marked with dashed line box. The enlarged images of the perturbed areas are included as the inset graphs (top right corner on (a–d)). The scale bar in (a) is applicable for (b) and in (c) is applicable for (d).

It is well known that SPs are sensitive to the environment [22, 23]. Here, we demonstrate that this platform can work as a sensor that can monitor the environmental changes in an area close to Ag NWs. On the bright-field image (Figure5(a)), a very small polystyrene (PS) nanoparticle with a diameter at about 40 nm is attached onto one side-wall of the Ag NW, which causes a bright spot appearing on L1 on the plasmonic image (Figure 5(b)). In Figure 5(c), we cannot identify obvious particles or something else from the area marked with dashed line box on the Ag NW. But for the same area in Figure 5(d), some bright spots appear on both L1 and L2. It means that there is real something on this Ag NW, which cannot be distinguished on the bright-field image. Therefore, if there is any perturbation or environmental change around the Ag NW, the propagating path will be distorted, which can be simultaneously imaged and positioned in the far-field camera. These results verify that this platform composing an Ag NW on a PBG structure is sensitive to the environment with the plasmonic leaky mode, which can be used to detect environmental changes around the Ag NW that cannot be distinguished in bright-field images.

## 3 Conclusion

In conclusion, due to the existence of the dielectric multilayer PBG structure, a plasmonic leaky mode can be sustained on an ultrathin Ag NW whose diameter is less than 100 nm. For this plasmonic leaky mode, the SPs will propagate along the two side-walls of this nanowire. Then, we can get two parallel plasmonic fields with spatial distance far below the diffraction limit. To the best of our knowledge, there are no reports on experimentally realizing two propagating parallel optical fields with a spatial distance less than 100 nm before. Both experiments and simulations show that, although their distance is far below the diffraction limit (∼242 nm) of the wide-field optical microscope used here, we can obverse two bright lines at the image plane. The mechanism is attributed to the unique phase distribution of the plasmonic fields on the two side-walls of the Ag NW. We also illustrate this platform as a nanosensor through locally perturbing the plasmonic fields with dielectric nanoparticles. What’s more, small environmental changes that cannot be observed in bright field image, can be clearly imaged in the plasmonic image. The experimental results show that the plasmonic leaky mode on Ag NWs can be used to detect environmental changes around the Ag NWs. Our work can obtain two plasmonic fields with a subdiffraction limited separation and realize the far-field optical imaging of them, which may find broad imaging and sensing applications.

## 4 Methods section

### 4.1 Experimental setup

A laser beam from a supercontinuum source (SuperK, NKT Photonics, and Denmark) was first coupled into a single-mode silica fiber with a nanometer-scale fiber taper. An acoustic-optical tunable filter was used to provide facile, flexible access to any wavelength in the SuperK spectrum. In our experiment, the wavelength was fixed at 590 nm. The fiber taper was controlled to be in contact with the Ag NW via a precise manipulator. The optical signals propagating along the nanowires were then imaged using a leakage radiation system setup with a high numerical aperture objective (100 X and NA of 1.49). The images in the far-field were recorded by a camera (Retiga 6000, Qimaging, Canada).

### 4.2 Samples preparation

The dielectric multilayers were fabricated via plasma-enhanced chemical vapor deposition (PECVD, Plasma Pro System 100, Oxford) of SiO_2_ and Si_3_N_4_ on a standard microscope cover glass (0.17 mm thickness) at a vacuum pressure of <0.1 mTorr and at a temperature of 300 °C. Here, SiO_2_ is the low refractive index dielectric and Si_3_N_4_ is the high refractive index dielectric. The thicknesses of these dielectric layers are 105 and 88 nm, respectively. In total 14 layers were deposited on top of each other. The thickness of the top SiO_2_ layer is approximately 160 nm. The Ag NWs were purchased from Nanjing XFNANO Materials Tech Co., Ltd., China. The synthesis steps of Ag NWs using wet-chemistry approaches are detailed in the study by Wei et al. [3]. The diameters of Ag NWs used in the main text are about 40 and 60 nm. There is a PVP cladding layer on the Ag NWs with a thickness of about 15 nm. The total diameters of the two Ag NWs are about 70 and 90 nm. The PVP cladding layer can make the Ag NWs stable for long-term use. The PS nanoparticles were bought from Thermo Fisher Scientific, USA, and their attachment to the Ag NW was realized by a MET One Aerosol Particle Generator 255 Gen.

### 4.3 Numerical simulations

The numerical simulations of the plasmonic mode (Figure 3) were performed with the finite element method (FEM) [24]. The numerical simulations of SPs propagating along an Ag NW (Figure 4) were performed with the finite-difference time-domain (FDTD) method [25], and the SPs were excited by a plane wave (total-field scattered-field source). All simulation parameters were chosen to match experimental conditions. The refractive index of silver was based on the experimental values [26]. The refractive index of the PVP cladding layer was set as 1.57 and its thickness was 15 nm. The refractive index of the air around the Ag NW was set as 1.

## Supplementary Material

Supplementary Material
