# Supplementary material for: Far-field optical imaging of surface plasmons with a subdiffraction limited separation
Source: Nanophotonics. Author manuscript; Available in PMC 2022 Mar 23. (PMC8942129; doi:10.1515/nanoph-2020-0500)
Supplement: Supplementary Material [file NIHMS1691514-supplement-Supplementary_Material.docx]

**Supplementary Material**

Far-Field Optical Imaging of Surface Plasmons with a Sub-Diffraction Limited Separation

Yifeng Xiang, Junxue Chen, Xi Tang, Ruxue Wang, Qiwen Zhan, Joseph R. Lakowicz and Douguo Zhang^*^

Yifeng Xiang: Key Laboratory of OptoElectronic Science and Technology for Medicine of Ministry of Education, Fujian Provincial Key Laboratory of Photonics Technology, College of Photonic and Electronic Engineering, Fujian Normal University, Fuzhou 350117, China

Junxue Chen: College of Science, Guilin University of Technology, Guilin 541004, China

Xi Tang: Institute of Photonics, Department of Optics and Optical Engineering, University of Science and Technology of China, Hefei, Anhui, 230026, China

Ruxue Wang: State Key Laboratory of Functional Materials for Informatics, Shanghai Institute of Microsystem and Information Technology, Chinese Academy of Sciences, Shanghai, 200050, China

Qiwen Zhan: Department of Electro-Optics and Photonics, University of Dayton, 300 College Park, Dayton, OH, 45469-2951, United States; and School of Optical-Electrical and Computer Engineering, University of Shanghai for Science and Technology, Shanghai, 200093, China

Joseph R. Lakowicz: Center for Fluorescence Spectroscopy, Department of Biochemistry and Molecular Biology, University of Maryland School of Medicine, 725 West Lombard St., Baltimore, MD 21201, United States.

*Corresponding author: Douguo Zhang, Institute of Photonics, Department of Optics and Optical Engineering, University of Science and Technology of China, Hefei, Anhui, 230026, China, E-mail: dgzhang@ustc.edu.cn

**SPs propagating along Ag NWs on a glass substrate**

When thin Ag NWs are placed on a glass substrate, the plasmonic leaky mode disappears and only the plasmonic bound mode exists. SPs propagating along the Ag NWs cannot leak photons into substrate and only the scattering light in the end of the Ag NWs can be observed.


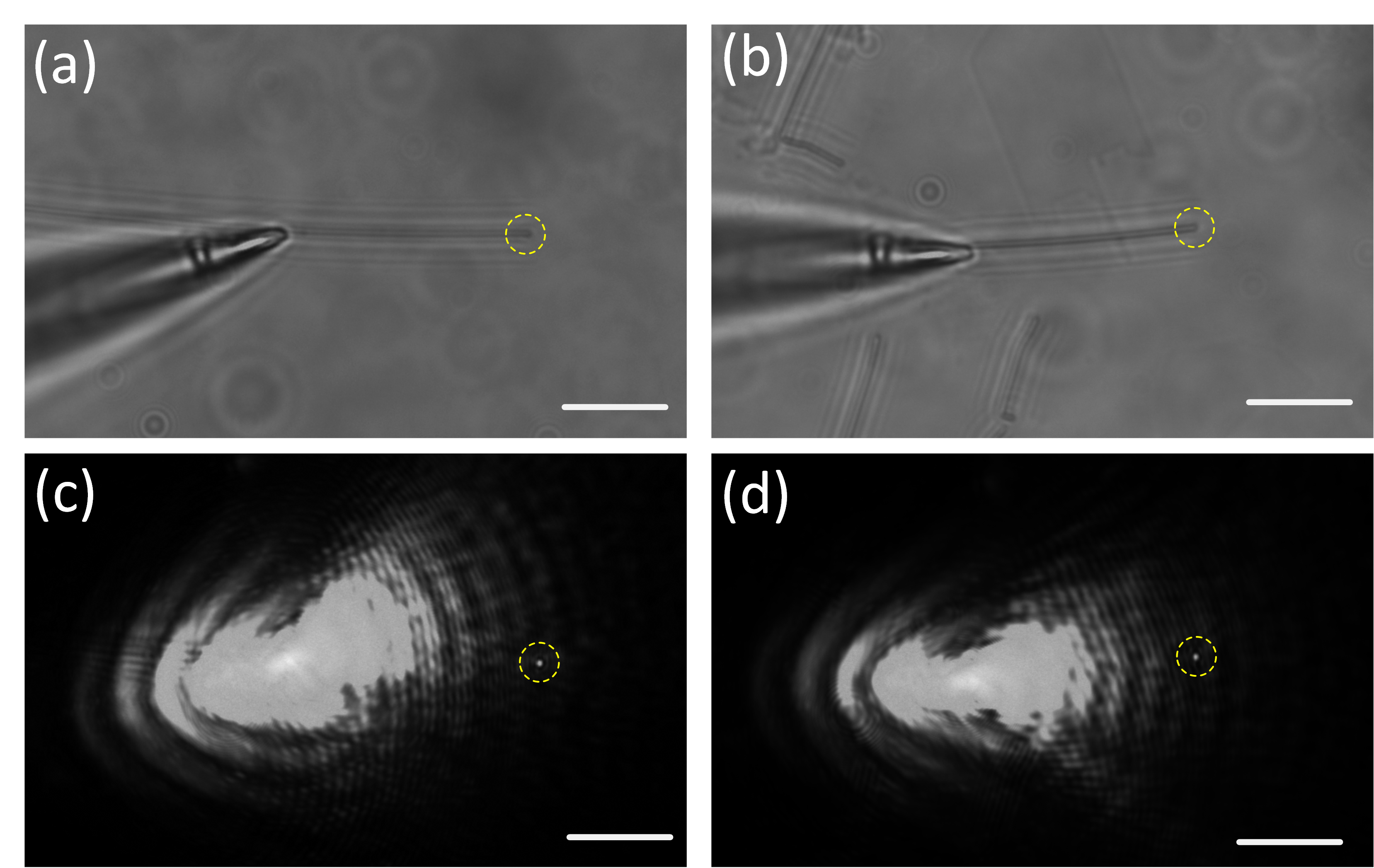


Figure S1. Images of SPs propagating along Ag NWs on a glass substrate in the far-field. The diameter of the Ag NW is about 90 nm in (a, c) and 120 nm in (b, d). (a, b) The bright field images of the Ag NW. (b, d) The corresponding dark field images for (a, b). The ends of Ag NWs are indicated by yellow dashed circles. The length of the scale bars is 5 μm.

**Images in the far-field with different incident wavelengths**

When the plasmonic mode is excited at the wavelength of 540 nm, both TE and TM polarization are in the forbidden band and the leakage radiation cannot not pass through the PBG structure. The SPs propagating along an Ag NW cannot be imaged in the far-field, as shown in Figure S2(b). When the incident wavelength is increased to 640 nm, the plasmonic mode is cut off by the light line and SPs cannot propagate along Ag NWs, as presented in Figure S2(d).


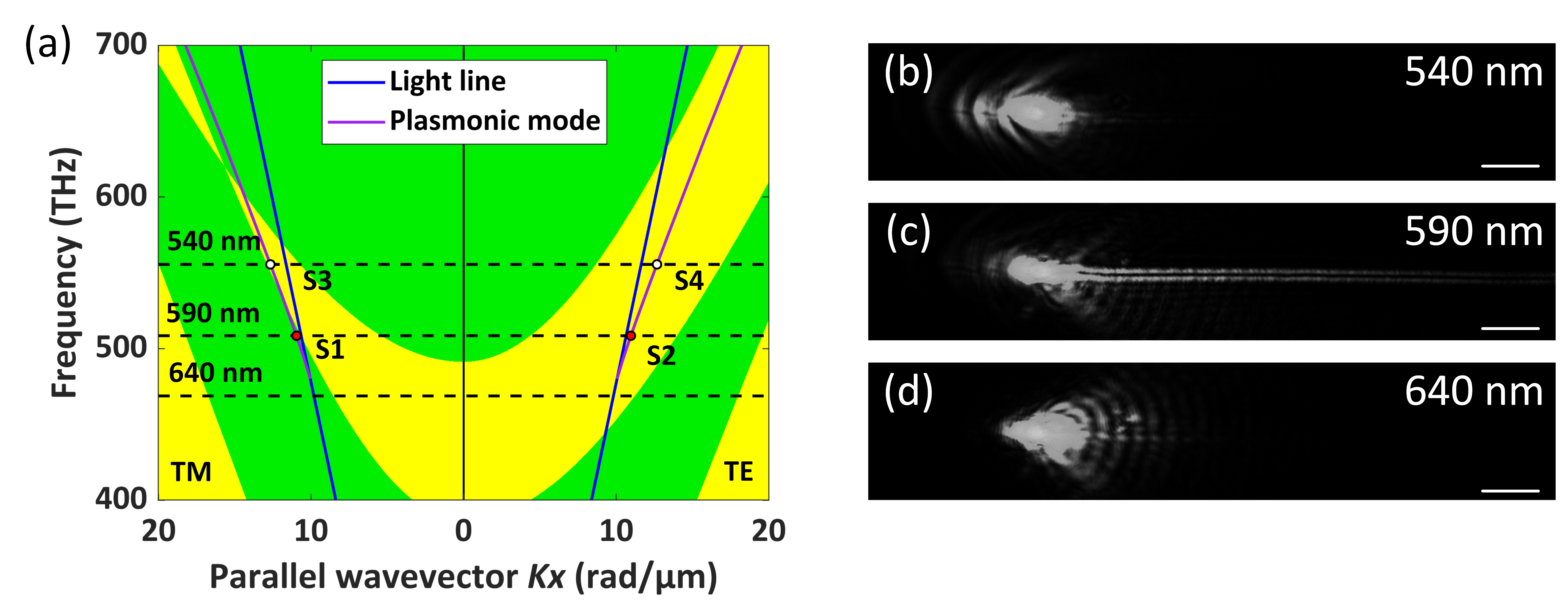


Figure S2. Images in the far-field with different incident wavelengths. (a) The white spots (S3 and S4) and the red spots (S1 and S2) on the dispersion curve for the plasmonic mode correspond to the excitation wavelength at 540 nm and 590 nm, respectively. The images in the far-field with the incident wavelength at (b) 540 nm, (c) 590 nm and (d) 640 nm.

**Simulated images in the far-field for different NA objectives**

For different NA objectives in the imaging system, the distance between the two bright lines in the image plane is different, which depends on the resolution ability of the imaging system. As the resolution of the imaging system increases, the image becomes clearer and the distance between the two lines decreases. When the NA of the objective is too small (NA = 1.15), the two bright lines in the image plane cannot be distinguished.





Figure S3. Images in the far-field for different NA objectives. The NA of the objective is 1.05 in (a, b), 1.15 in (c, d), 1.25 in (e, f), and 1.35 in (g, h). (a, c, e, g) The electric field intensity distributions at the image plane. (b, d, f, h) The corresponding average intensity profiles perpendicular to the Ag NW in area between the white dashed lines in (a, c, e, g). Two bright lines cannot be distinguished in (a). The distance between two peaks is 720 nm in (d), 576 nm in (f), and 480 nm in (h).

**The PBG of a periodic structure**

A schematic of an infinite binary PBG structure is shown in Figure S4. For the case of TE polarization, the characteristic matrix of each single layer is

$\begin{aligned} M=\left( \begin{matrix} \cos\left( pd \right) & \frac{i}{p}\sin\left( pd \right) \\ ip\sin\left( pd \right) & \cos\left( pd \right) \end{matrix} \right),\#\left( S1 \right) \end{aligned}$

where $p=\sqrt{\varepsilon k_{0}^{2}-\beta^{2}}$, $\beta$ denotes the wave vector projected on the $x$ axis, and $\varepsilon$ and $d$ represent the permittivity and thickness of the single layer, respectively. The characteristic matrix of a unit cell consisting of two single layers is the product of the two individual characteristic matrices:

$\begin{aligned} M_{uv}=M_{u}M_{v}=\left( \begin{matrix} m_{11} & m_{12} \\ m_{21} & m_{22} \end{matrix} \right),\#\left( S2 \right) \end{aligned}$

where

$\begin{aligned} \begin{aligned} \#m_{11}=\cos\left( p_{u}d_{u} \right)\cos\left( p_{v}d_{v} \right)-\frac{p_{u}}{p_{v}}\cos\left( p_{u}d_{u} \right)\cos\left( p_{v}d_{v} \right), \\ \#m_{12}=\frac{i}{\beta_{v}}\cos\left( p_{u}d_{u} \right)\cos\left( p_{v}d_{v} \right)+\frac{i}{\beta_{u}}\cos\left( p_{u}d_{u} \right)\cos\left( p_{v}d_{v} \right), \\ \#m_{21}=i\beta_{v}\cos\left( p_{u}d_{u} \right)\cos\left( p_{v}d_{v} \right)+i\beta_{u}\cos\left( p_{u}d_{u} \right)\cos\left( p_{v}d_{v} \right), \\ \#m_{22}=\cos\left( p_{u}d_{u} \right)\cos\left( p_{v}d_{v} \right)-\frac{p_{v}}{p_{u}}\cos\left( p_{u}d_{u} \right)\cos\left( p_{v}d_{v} \right). \end{aligned}\#\left( S3 \right) \end{aligned}$

The electromagnetic wave propagating in periodic medium satisfies the periodic condition of the Bloch wave, which can be expressed as

$\begin{aligned} M_{uv}=\left( \begin{matrix} m_{11} & m_{12} \\ m_{21} & m_{22} \end{matrix} \right)=e^{iK\Lambda},\#\left( S4 \right) \end{aligned}$

where $K$ is the wave vector of the Bloch wave and $\Lambda=d_{u}+d_{v}$. The dispersion relation for the Bloch wave can be written as

$\begin{aligned} \cos\left( K\Lambda\right)=\cos\left( p_{u}d_{u} \right)\cos\left( p_{v}d_{v} \right)-\frac{1}{2}\left( \frac{p_{v}}{p_{u}}+\frac{p_{u}}{p_{v}} \right)\sin\left( p_{u}d_{u} \right)\sin\left( p_{v}d_{v} \right).\#\left( S5 \right) \end{aligned}$

Regimes where $\left| \cos\left( K\Lambda\right) \right|<1$ correspond to the real $K$ and thus to propagating Bloch waves. When $\left| \cos\left( K\Lambda\right) \right|>1$, $K$ has an imaginary part and the Bloch wave is evanescent, which correspond to forbidden bands for the periodic medium. Since $\frac{1}{2}\left( \frac{p_{v}}{p_{u}}+\frac{p_{u}}{p_{v}} \right)>1$, $\cos\left( K\Lambda\right)<-1$ is satisfied when $p_{u}d_{u}\sim p_{v}d_{v}\sim\frac{\pi}{2}$. This relation can be used to design the initial parameters of a PBG structure.

For the case of TM polarization, the dispersion relation of the Bloch wave is given by

$\begin{aligned} \cos\left( K\Lambda\right)=\cos\left( p_{u}d_{u} \right)\cos\left( p_{v}d_{v} \right)-\frac{1}{2}\left( \frac{p_{v}\varepsilon_{u}}{p_{u}\varepsilon_{v}}+\frac{p_{u}\varepsilon_{v}}{p_{v}\varepsilon_{u}} \right)\sin\left( p_{u}d_{u} \right)\sin\left( p_{v}d_{v} \right).\#\left( S6 \right) \end{aligned}$


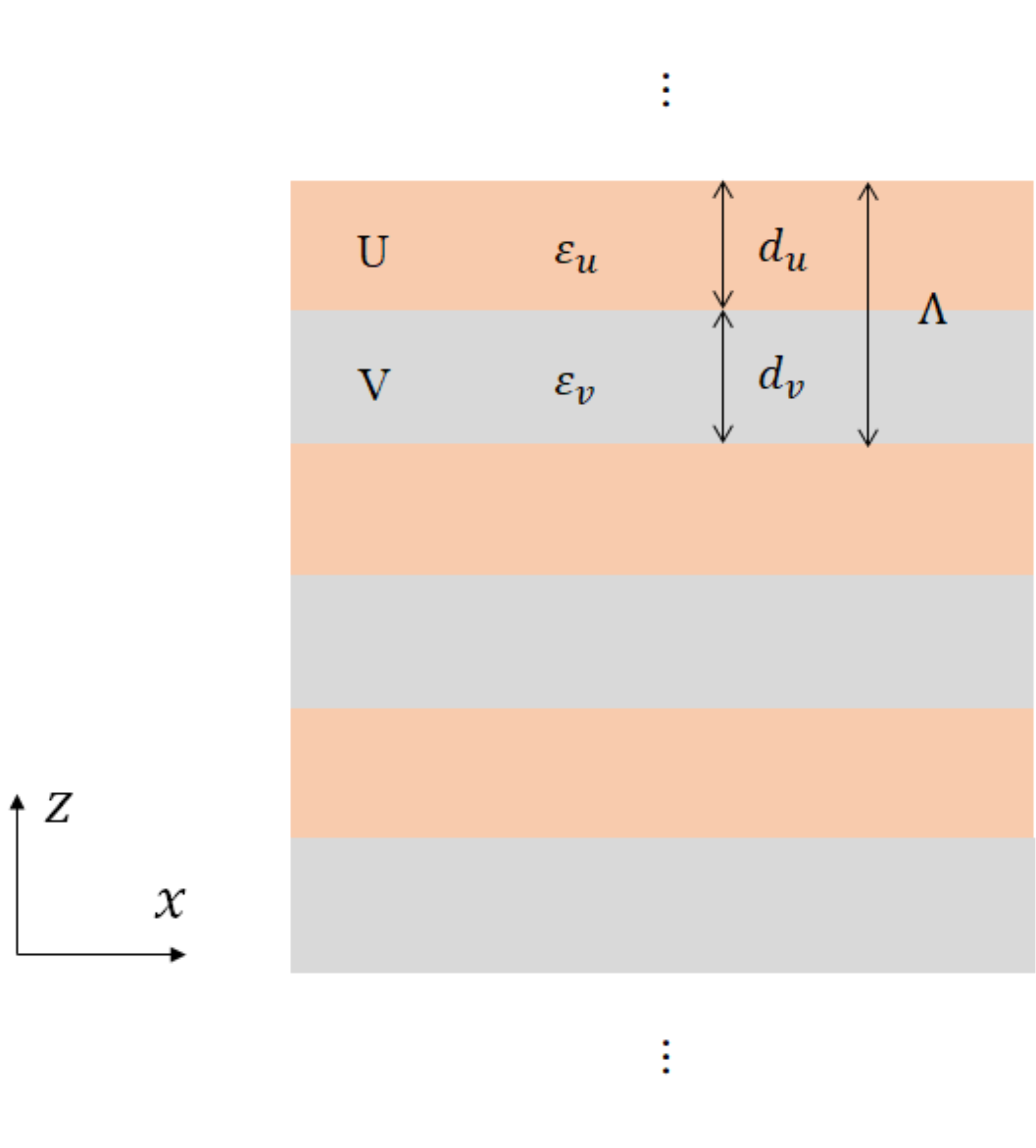


Figure S4. An infinite binary PBG structure. The periodic structure with a unit cell comprises two layers with thickness $d_{u}$ and $d_{v}$ and permittivity $\varepsilon_{u}$ and $\varepsilon_{v}$, respectively.

**Coherent imaging theory**

A schematic of the LRM is shown in Figure S5. Due to the high NA objective in the LRM, the vector diffraction theory is used to calculate light propagation through the imaging system [1]. Here, the objective satisfies the “sine” condition and the mathematical treatment of the objective as a black box does not actually consider the physical light propagation in the objective.

First, we calculate the electric field distribution at the far-field projection plane. The light originating from the object plane $\Pi$ propagates to the sphere $\Sigma_{1}$. The electric field $\boldsymbol{E}_{\Sigma_{1}}$ on $\Sigma_{1}$ can be calculated by

$$\begin{aligned} \boldsymbol{E}_{\Sigma_{1}}\left( \boldsymbol{x}_{\mathbf{1}} \right)=\frac{nk_{0}}{i2\pi}\iint_{\Pi} \boldsymbol{E}_{\Pi}\left( \boldsymbol{x} \right)\frac{\exp\left( ink_{0}R_{1} \right)}{R_{1}}\cos\theta_{1}d^{2}\boldsymbol{x},\#\left( S7 \right) \end{aligned}$$

where $R_{1}=\left| \boldsymbol{X-}\boldsymbol{X}_{\boldsymbol{1}} \right|$ is the distance between the point on $\Pi$ (with coordinates $\boldsymbol{X}=\left[ \boldsymbol{x},z \right]$) and the point on $\Sigma_{1}$ (with cooridnates $\boldsymbol{X}=\left[ \boldsymbol{x}_{1},z_{1} \right]$), and the angel $\theta_{1}$ is defined by $\cos\theta_{1}=(z_{1}-z)/f$. Following the approximation $\left| \boldsymbol{x} \right|\ll R_{1}\sim f$, the Green function can be simplified to

$$\begin{aligned} \frac{\exp\left( ink_{0}R_{1} \right)}{R_{1}}\approx\frac{\exp\left( ink_{0}f \right)}{f}\exp\left( -\frac{ink_{0}\boldsymbol{x\cdot}\boldsymbol{x}_{\boldsymbol{1}}}{f} \right).\#\left( S8 \right) \end{aligned}$$

So $\boldsymbol{E}_{\Sigma_{1}}$ can be expressed as

$$\begin{aligned} \boldsymbol{E}_{\Sigma_{1}}\left( \boldsymbol{x}_{\boldsymbol{1}} \right)=\frac{\cos\theta_{1}nk_{0}\exp\left( ik_{0}nf \right)}{i2\pi f}\iint_{\Pi} \boldsymbol{E}_{\Pi}\left( \boldsymbol{x} \right)\exp\left( -i\boldsymbol{k\cdot x} \right)d^{2}\boldsymbol{x},\#\left( S9 \right) \end{aligned}$$

where $\boldsymbol{k=}nk_{0}\boldsymbol{x}_{\boldsymbol{1}}\boldsymbol{/}f$. Then, the spherical wave front $\Sigma_{1}$ evolves into a planar wave front $\Pi_{1}$ after traveling through the objective. Using the energy conservation, we get

$$\begin{aligned} \left| \boldsymbol{E}_{\Pi_{1}}\left( \boldsymbol{x}_{\boldsymbol{1}} \right) \right|^{2}d^{2}\boldsymbol{x}_{\boldsymbol{1}}=n\left| \boldsymbol{E}_{\Sigma_{1}}\left( \boldsymbol{x}_{\boldsymbol{1}} \right) \right|^{2}dS_{1},\#\left( S10 \right) \end{aligned}$$

where $dS_{1}$ is the surface element on $\Sigma_{1}$ and has the relation $\cos\theta_{1}dS_{1}=d^{2}\boldsymbol{x}_{1}$. Taking into account the vector direction of electromagnetic field, the electric field is written after separation into TM and TE polarization as

$$\begin{aligned} \boldsymbol{E}_{\Pi_{1}}\left( \boldsymbol{x}_{\boldsymbol{1}} \right)=\sqrt{\frac{n}{\cos\theta_{1}}}\left\{ \left[ \boldsymbol{E}_{\Sigma_{1}}\left( \boldsymbol{x}_{\boldsymbol{1}} \right)\cdot{\hat{\boldsymbol{\theta}}}_{1} \right]{\hat{\boldsymbol{\rho}}}_{1}+\left[ \boldsymbol{E}_{\Sigma_{1}}\left( \boldsymbol{x}_{\boldsymbol{1}} \right)\cdot{\hat{\boldsymbol{\varphi}}}_{1} \right]{\hat{\boldsymbol{\varphi}}}_{1} \right\}P_{NA}\left( \rho_{1} \right),\#\left( S11 \right) \end{aligned}$$

where ${\hat{\boldsymbol{\rho}}}_{1}$ and ${\hat{\boldsymbol{\varphi}}}_{1}$ are the radial and angular unit vectors on $\Pi_{1}$ respectively, and ${\hat{\boldsymbol{\theta}}}_{1}=cos\theta_{1}{\hat{\boldsymbol{\rho}}}_{1}-\cos\theta_{1}\hat{\boldsymbol{z}}$. In this formula, $P_{NA}(\rho_{1})$ is the pupil function of the objective and $\rho_{1}=\left| \boldsymbol{x}_{1} \right|=f\sin\theta_{1}$. $P_{NA}\left( \rho_{1} \right)=1$ if $\rho_{1}\leq f\cdot NA/n$ and $P_{NA}\left( \rho_{1} \right)=0$ otherwise. $\Pi_{1}$ can be regarded as the far-field projection plane. The electric field on $\Pi_{1}$ can be written as

$$\begin{aligned} \boldsymbol{E}_{\Pi_{1}}\left( \boldsymbol{x}_{\boldsymbol{1}} \right)=N\sqrt{\cos\theta_{1}}\left\{ \left[ {\tilde{\boldsymbol{E}}}_{\Pi}\left( \boldsymbol{k} \right)\cdot{\hat{\boldsymbol{\theta}}}_{1} \right]{\hat{\boldsymbol{\rho}}}_{1}+\left[ {\tilde{\boldsymbol{E}}}_{\Pi}\left( \boldsymbol{k} \right)\cdot{\hat{\boldsymbol{\varphi}}}_{1} \right]{\hat{\boldsymbol{\varphi}}}_{1} \right\}P_{NA}\left( \rho_{1} \right),\#\left( S12 \right) \end{aligned}$$

where $N=\frac{\sqrt{n^{3}}k_{0}\exp\left( ik_{0}nf \right)}{i2\pi f}$ and ${\tilde{\boldsymbol{E}}}_{\Pi}\left( \boldsymbol{k} \right)$ is the bidimensional Fourier transform of the field $\boldsymbol{E}_{\Pi}\left( \boldsymbol{x} \right)$ defined as ${\tilde{\boldsymbol{E}}}_{\Pi}\left( \boldsymbol{k} \right)=\iint_{\Pi} \boldsymbol{E}_{\Pi}\left( \boldsymbol{x} \right)\exp\left( -i\boldsymbol{k\cdot x} \right)d^{2}\boldsymbol{x}$.

The next step is to calculate that the light propagates from the far-field projection plane to the image plane. The propagation between the objective ($\Pi_{1}$) and the tube lens ($\Pi_{2}$) can be treated in the paraxial approximation ($\left| \boldsymbol{x}_{1} \right|{=\rho}_{1}\ll L$ and $|\boldsymbol{x}_{2}|{=\rho}_{2}\ll L$). It is possible to assume $\cos\theta\approx1$. The field $\boldsymbol{E}_{\Pi_{2}}\left( \boldsymbol{x}_{\boldsymbol{2}} \right)$ in the plane $\Pi_{2}$ be calculated by

$$\begin{aligned} \boldsymbol{E}_{\Pi_{2}}\left( \boldsymbol{x}_{2} \right)=\frac{k_{0}\exp\left( ik_{0}L \right)}{2\pi iL}\exp\left( \frac{ik_{0}{\rho_{2}}^{2}}{2L} \right)\iint_{\Pi_{1}} \boldsymbol{E}_{\Pi_{1}}\left( \boldsymbol{x}_{1} \right)\exp\left( \frac{ik_{0}{\rho_{1}}^{2}}{2L} \right)\exp\left( -\frac{ik_{0}\boldsymbol{x}_{1}\boldsymbol{\cdot}\boldsymbol{x}_{\boldsymbol{2}}}{2L} \right)d^{2}\boldsymbol{x}_{1}.\#\left( S13 \right) \end{aligned}$$

The propagation through the tube lens is similar to the one for the objective. In practical calculation, $\theta_{2}\approx0$ can be obtained since $\rho_{2}\ll f^{'}$. The field $\boldsymbol{E}_{\Sigma_{2}}\left( \boldsymbol{x}_{2} \right)$ on $\Sigma_{2}$ is given by

$$\begin{aligned} \boldsymbol{E}_{\Sigma_{2}}\left( \boldsymbol{x}_{2} \right)=\boldsymbol{E}_{\Pi_{2}}\left( \boldsymbol{x}_{2} \right)P_{{NA}^{'}}\left( \rho_{2} \right).\#\left( S14 \right) \end{aligned}$$

Finally, the light from the sphere $\Sigma_{2}$ is focused on the image plane $\Pi^{'}$. In the approximation of $\left| \boldsymbol{x}^{'} \right|=\rho^{'}\ll f^{'}$ and $\theta_{2}\approx0$, the field $\boldsymbol{E}_{\Pi^{'}}\left( \boldsymbol{x}_{\mathbf{2}} \right)$ in the plane $\Pi^{'}$ can be obtained by

$$\begin{aligned} \boldsymbol{E}_{\Pi^{'}}(\boldsymbol{x}^{'}))=\frac{k_{0}\exp\left( ik_{0}f^{'} \right)\exp\left( \frac{ik_{0}{\rho^{'}}^{2}}{2f^{'}} \right)}{2\pi if^{'}}\iint_{\Pi_{2}} \boldsymbol{E}_{\Sigma_{2}\text{ }}\left( \boldsymbol{x}_{2} \right)\exp\left( -\frac{ik_{0}\boldsymbol{x}_{2}\cdot\boldsymbol{x}^{'}}{2f^{'}} \right)d^{2}\boldsymbol{x}_{\boldsymbol{2}}.\#\left( S15 \right) \end{aligned}$$

Using equation (S12), (S13) and (S14) leads to

$$\begin{aligned} \begin{matrix} \boldsymbol{E}_{\Pi^{'}}\left( \boldsymbol{x}^{'} \right) \\ =\frac{-{k_{0}}^{2}\exp\left( ik_{0}f^{'} \right)\exp\left( \frac{ik_{0}{\rho^{'}}^{2}}{2f^{'}} \right)\exp\left( ik_{0}L \right)}{4\pi^{2}f^{'}L}\iint_{\Pi_{1}} \boldsymbol{E}_{\Pi_{1}\text{ }}\left( \boldsymbol{x}_{\boldsymbol{2}} \right)\exp\left( \frac{ik_{0}{\rho_{1}}^{2}}{2L} \right)I\left( \boldsymbol{x}_{\boldsymbol{1}},\boldsymbol{x}^{\boldsymbol{'}} \right)d^{2}\boldsymbol{x}_{\boldsymbol{1}}, \end{matrix}\#\left( S16 \right) \end{aligned}$$

where

$$\begin{aligned} I\left( \boldsymbol{x}_{\boldsymbol{1}},\boldsymbol{x}^{\boldsymbol{'}} \right)=\iint_{\Pi_{2}} P_{{NA}^{'}}\left( \rho_{2} \right)\exp\left( -\frac{ik_{0}\boldsymbol{x}_{2}\cdot\boldsymbol{x}^{'}}{2f^{'}} \right)\exp\left( \frac{ik_{0}{\rho_{2}}^{2}}{2L} \right)\exp\left( -\frac{ik_{0}\boldsymbol{x}_{1}\boldsymbol{\cdot}\boldsymbol{x}_{\boldsymbol{2}}}{L} \right)d^{2}\boldsymbol{x}_{\boldsymbol{2}}.\#(S17) \end{aligned}$$

In the imaging system, the radius of the collimated beam propagating from $\Pi_{1}$ to $\Pi_{2}$ is approximately given by the radius $r_{1}$ of the exit pupil of the objective, which is much smaller than the tube lens radius $r_{2}$. It is possible to assume that $P_{{NA}^{'}}\left( \rho_{2} \right)=1$ over the interest region in the plane $\Pi_{2}$ and the integrate range of $\boldsymbol{x}_{2}$ is $-\infty$ to +$\infty$ along the $x_{2}$ and $y_{2}$ directions. $\boldsymbol{E}_{\Pi^{'}}(\boldsymbol{x}^{'})$ can be simplified to

$$\begin{aligned} \boldsymbol{E}_{\Pi^{'}}\left( \boldsymbol{x}^{'} \right)=N^{'}\iint_{\Pi_{1}} \boldsymbol{E}_{\Pi_{1}\text{ }}\left( \boldsymbol{x}_{1} \right)\exp\left( -i\boldsymbol{k}\cdot\frac{\boldsymbol{x}^{'}}{M} \right)d^{2}\boldsymbol{x}_{\boldsymbol{1}},\#\left( S18 \right) \end{aligned}$$

where $N^{'}=\frac{k_{0}}{i2\pi f^{'}}\exp\left( ik_{0}\left( f^{'}+\triangle\right) \right)\exp\left( \frac{ik_{0}{\rho^{'}}^{2}}{2f^{'}}\left( 1-\frac{\triangle}{f^{'}} \right) \right)$ and $M=nf^{'}/f$ is the magnification of the microscope. The field $\boldsymbol{E}_{\Pi^{'}}(\boldsymbol{x}^{'})$ in the image plane can be calculated by Fourier transformation of the field $\boldsymbol{E}_{\Pi_{1}\text{ }}\left( \boldsymbol{x}_{1} \right)$ in the far-field projection plane.


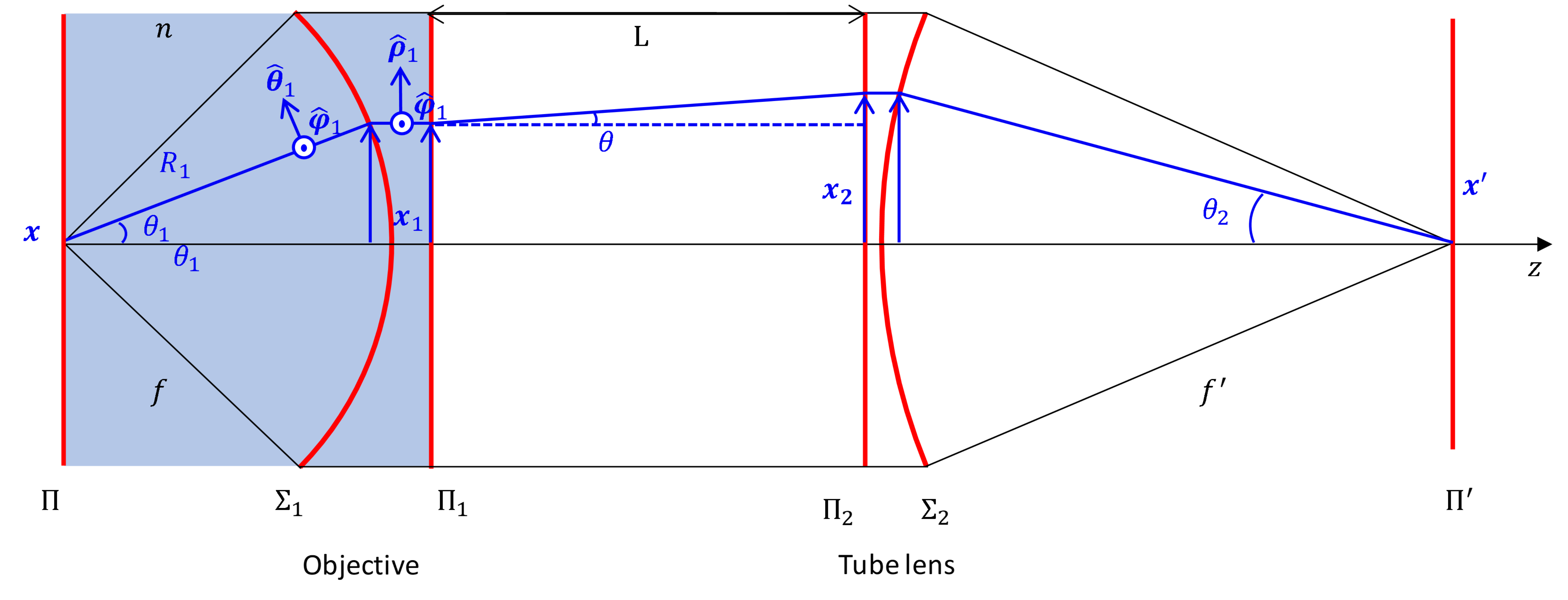


Figure S5. Schematic of the LRM with a high NA objective. Light originating from plane $\Pi$ is collimated along the optical axis after crossing the reference sphere $\Sigma_{1}$. $\Pi_{1}$ is the back focal plane of the objective. $\Pi_{2}$ and $\Sigma_{2}$ play for the tube lens the same role played by $\Pi_{1}$ and $\Sigma_{1}$ for the objective. The objective plane $\Pi$ is mapped onto the image plane $\Pi^{'}$. The refractive indices of the oil used in the objective and the environment are set as $n$ and 1, respectively. The focal lengths of the objective and the tube lens are $f$ and $f^{'}$, respectively.

**Reference**

1. M. Berthel, Q. B. Jiang, C. Chartrand, J. Bellessa, S. Huant, C. Genet, A. Drezet. Coherence and aberration effects in surface plasmon polariton imaging. Phys Rev E 2015;92:033202.
